# Screening, diagnosis and follow-up of Brugada syndrome in children: a Dutch expert consensus statement

**DOI:** 10.1007/s12471-022-01723-6

**Published:** 2022-10-12

**Authors:** P. J. Peltenburg, Y. M. Hoedemaekers, S. A. B. Clur, N. A. Blom, A. C. Blank, E. P. Boesaard, S. Frerich, F. van den Heuvel, A. A. M. Wilde, J. A. E. Kammeraad

**Affiliations:** 1 Department of Paediatric Cardiology, Emma Children’s Hospital, Amsterdam University Medical Centres, University of Amsterdam, Amsterdam, The Netherlands; 2 Amsterdam University Medical Centres, Heart Centre; Department of Clinical and Experimental Cardiology, Amsterdam Cardiovascular Sciences, University of Amsterdam, Amsterdam, The Netherlands; 3 Department of Clinical Genetics, Radboud University Medical Centre, Nijmegen, The Netherlands; 4 Department of Paediatric Cardiology, Willem-Alexander Children’s Hospital, Leiden University Medical Centre, Leiden, The Netherlands; 5 Department of Paediatric Cardiology, Wilhelmina Children’s Hospital, Utrecht University Medical Centre, Utrecht, The Netherlands; 6 Department of Paediatric Cardiology, Amalia Children’s Hospital, Radboud University Medical Centre, Nijmegen, The Netherlands; 7 Department of Paediatric Cardiology, Maastricht University Medical Centre, Maastricht, The Netherlands; 8 Department of Paediatric Cardiology, University Medical Centre Groningen, Groningen, The Netherlands; 9 Department of Paediatric Cardiology, Erasmus Medical Centre-Sophia Children’s Hospital, Rotterdam, The Netherlands

**Keywords:** Brugada syndrome, DNA diagnostics, Family screening, Follow-up, Children

## Abstract

Brugada syndrome (BrS) is a rare inherited arrhythmia syndrome. Affected children may experience life-threatening symptoms, mainly during fever. The percentage of *SCN5A* variant carriers in children is higher than in adults. Current diagnostic and follow-up policies for children with (a family history of) BrS vary between centres. Here, we present a consensus statement based on the current literature and expert opinions to standardise the approach for all children with BrS and those from BrS families in the Netherlands. In summary, BrS is diagnosed in patients with a spontaneous type 1 electrocardiogram (ECG) pattern or with a Shanghai score ≥ 3.5 including ≥ 1 ECG finding. A sodium channel-blocking drug challenge test should only be performed after puberty with a few exceptions. A fever ECG is indicated in children with suspected BrS, in children with a first-degree family member with definite or possible BrS according to the Shanghai criteria with a *SCN5A *variant and in paediatric *SCN5A *variant carriers. In-hospital rhythm monitoring during fever is indicated in patients with an existing type 1 ECG pattern and in those who develop such a pattern. Genetic testing should be restricted to *SCN5A*. Children with BrS and children who carry an *SCN5A* variant should avoid medication listed at www.brugadadrugs.org and fever should be suppressed. Ventricular arrhythmias or electrical storms should be treated with isoproterenol infusion.

**Supplementary Information:**

The online version of this article (10.1007/s12471-022-01723-6) contains supplementary material, which is available to authorized users.

## Introduction

Brugada syndrome (BrS) is a rare inherited arrhythmia syndrome causing conduction abnormalities, bradyarrhythmias and tachyarrhythmias. Although the prevalence of BrS is lower in children than in adults, their risk of developing symptoms is higher, especially during fever [1, 2]. Since the initial report on BrS in 1992, which included three children with recurrent aborted cardiac arrest (ACA),[3] research into this syndrome has evolved and its clinical management has improved [4]. However, the current practice of cardiologists, paediatric cardiologists and clinical geneticists with regard to the diagnosis and follow-up of BrS in children varies between clinical centres, which could lead to different strategies within the same family.

We therefore aimed to provide a Dutch expert consensus statement on the diagnosis, family screening and follow-up of paediatric BrS, ensuring the same standard of care for all children with BrS and for BrS families in the Netherlands. Our recommendations are based on the literature, expert consensus meetings and the experiences of the contributing authors and have been approved by all authors.

## Diagnosis of Brugada syndrome in children

### Electrocardiogram

BrS is diagnosed in patients with a revised Shanghai score ≥ 3.5 (Tab. 1; [4]). This means that the diagnosis is made in children with a spontaneous type 1 pattern on a standard electrocardiogram (ECG) (see Figure S1 in Electronic Supplementary Material) or on a higher right precordial lead ECG (Brugada ECG) (see Figure S2 in Electronic Supplementary Material). The Brugada ECG increases the likelihood of finding a type 1 pattern [5]. Fever can trigger a type 1 ECG pattern [6, 7]. When accompanied by other characteristics, a fever-induced type 1 pattern also leads to the diagnosis of BrS [4].

**Table 1:** Shanghai Score System for diagnosing BrS^a^

Factor	Points
*ECG (12-lead/ambulatory)*^b^	
Spontaneous type 1 Brugada pattern on standard or Brugada ECG	3.5
Fever-induced type 1 Brugada pattern on standard or Brugada ECG	3
Type 2 or 3 Brugada ECG pattern that converts with sodium channel-blocking drug challenge	2
*Clinical history*^a^	
Unexplained cardiac arrest or documented ventricular fibrillation/polymorphic ventricular tachycardia	3
Nocturnal agonal respirations	2
Suspected arrhythmic syncope	2
Syncope of unclear mechanism or unclear aetiology	1
Atrial flutter or fibrillation in patients < 30 years without alternative aetiology	0.5
*Family history*^a^	
First- or second-degree relative with definite BrS	2
Suspicious SCD (during fever, at night, or when taking Brugada-aggravating drugs) in a first- or second-degree relative	1
Unexplained SCD < 45 years in a first- or second-degree relative with negative autopsy	0.5
*Genetic test result*	
Probable pathogenic mutation in BrS susceptibility gene	0.5
Total score (requires ≥ 1 ECG finding)	
≥ 3.5 points	Probable or definite BrS
2–3 points	Possible BrS
< 2 points	Non-diagnostic

*BrS* Brugada syndrome,* ECG* electrocardiogram, *SCD* sudden cardiac death

^a^ This table was adapted from an original table as reported by Antzelevitch et al. [4]

^b^ Only award points once for highest score within each category

### Sodium channel-blocking drug challenge test

Two cohort studies have shown that 20–24% of BrS-suspected children develop a type 1 pattern,[8, 9] whilst 3–7% have potentially life-threatening ventricular arrhythmias during a drug challenge test [9–11]. However, in a group of 53 asymptomatic children with an initial negative drug challenge test result, 23% had a positive test result when the drug challenge was repeated after puberty, which led to the BrS diagnosis [12].

Due to the low negative predictive value of the drug challenge test before puberty, a fever ECG is needed in children suspected of having BrS, irrespective of their response to the drug challenge test. Therefore, a drug challenge test before puberty is not recommended in BrS screening but might be indicated in exceptional cases, for example in children with ACA of unknown cause.

### Genetic testing

In 1998, Chen et al. described an association between BrS and the *SCN5A *gene [13]. Pathogenic variants of *SCN5A *have been found in 15–20% of BrS patients and in 40% of familial BrS cases [14, 15]. However, in paediatric BrS probands, the proportion of *SCN5A* variant carriers is higher (47–67%) [2, 16].

In children diagnosed with BrS, genetic testing solely for *SCN5A* and variant calling according to the American College of Medical Genetics guidelines are recommended [17]. All other genes have been demoted to ‘limited evidence of pathogenicity’ [18].

Presymptomatic genetic testing of a (likely) pathogenic familial *SCN5A* variant is recommended for children of carriers of a (likely) pathogenic *SCN5A* variant. The genetic test result will guide the recommendations for further follow-up.

### Clinical suspicion

BrS should be considered as a diagnosis in children with recurrent syncope, ACA or sudden cardiac death, especially if this occurs during fever [1]. If BrS is being considered, the child should be screened by a paediatric cardiologist by using standard and Brugada ECGs at rest and during fever (see Figure S2 in Electronic Supplementary Material).

## Family screening

**Fig. 1:**
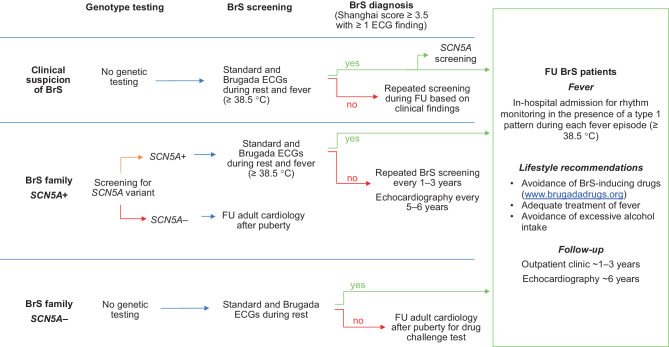
Flowchart for diagnosis and follow-up of Brugada syndrome (*BrS*) in children. A Brugada electrocardiogram (*ECG*) is an ECG recording for which the right precordial leads are positioned higher. *FU* follow-up

As BrS is mainly diagnosed in the third or fourth decade of life, most children are identified through family cascade screening [2]. Children with a first-degree relative with BrS should be screened according to the recommendations described below, which are summarised in Fig. 1.

### Families with a (likely) pathogenic SCN5A variant

In families with a (likely) pathogenic *SCN5A* variant causing BrS, consultation with a clinical geneticist—potentially during pregnancy—is advised to discuss presymptomatic testing of the familial *SCN5A *variant in the child. After delivery, a noninvasive umbilical cord blood sample can be taken to screen for the *SCN5A* variant. If the parents decide not to have their child undergo this screening, the same recommendations apply as for *SCN5A* variant carriers, as described below.

*SCN5A* variant-positive children should be evaluated by a paediatric cardiologist preferably before the first vaccination, shortly after the *SCN5A* variant screening test or during their first fever episode. During this visit, standard and Brugada ECG recordings are recommended. In addition, the child should be evaluated during fever (≥ 38.5 °C) with standard and Brugada ECGs every 3 years or more frequently if their body temperature is higher than it was during the previously recorded fever ECG [7]. If no abnormalities are seen, an outpatient visit once every 3 years, including standard and Brugada ECG recordings, is recommended.

Caution is warranted for the use of certain drugs (see www.brugadadrugs.org). If a particular drug cannot be avoided, an ECG recording to evaluate its effect should be considered. Echocardiography is recommended every 6 years due to the possible association of BrS with cardiomyopathy [19]. A 24-hour Holter monitoring during puberty is recommended on indication to look for signs of sinus node dysfunction, and an exercise test may be considered. A drug challenge test is contra-indicated.

Adult family members who are non-carriers of the familial *SCN5A *variant may still be at risk of BrS [20]. However, the prevalence is low, and the risk of symptoms is even lower. We therefore do not recommend BrS screening during childhood in *SCN5A *variant-negative children from *SCN5A* variant-positive families.

### Families without a (likely) pathogenic SCN5A variant

For children from BrS families without a (likely) pathogenic *SCN5A* variant, genetic testing is not meaningful. In fact, in a retrospective cohort study of 97 children with an a priori risk of BrS—based on family history of BrS or a BrS genotype—no children from *SCN5A* variant-negative families developed a type 1 ECG pattern during fever [7]. Therefore, we recommend evaluating children with a first-degree family member with definite or possible BrS according to the Shanghai criteria but without a pathogenic *SCN5A *variant only once. In these children, the paediatric cardiologist should evaluate their standard and Brugada ECGs after birth or after BrS has been diagnosed in a first-degree family member. If no abnormalities are found, further screening for BrS during childhood is not indicated. The child should be re-evaluated after puberty and a drug challenge test should be considered to definitively make or reject a BrS diagnosis.

For children with an abnormal BrS screening test, follow-up by a paediatric cardiologist is recommended. In families with a clear familial disease pattern without a (likely) pathogenic variant, children should be evaluated as being *SCN5A *variant carriers.

## Follow-up of children with Brugada syndrome

Children diagnosed with BrS are at risk for life-threatening arrhythmic events, especially during fever [1]. As there is currently no available therapy to prevent this, the follow-up focuses on risk stratification and lifestyle recommendations to minimise this risk [21, 22]. We recommend evaluation by a paediatric cardiologist or electrophysiologist once every 1–3 years with standard and Brugada ECGs. Echocardiography should be performed once every 6 years. A 24-hour Holter recording, an exercise-stress test and an implantable loop recorder may be considered, particularly to evaluate symptoms such as syncope and palpitations.

### Lifestyle recommendations

In children diagnosed with BrS, fever should be suppressed with acetaminophen with or without ibuprofen. Vaccinations should also be accompanied by acetaminophen treatment. Influenza vaccinations are not indicated.

During fever, standard and Brugada ECG recordings are indicated. If a type 1 pattern is pre-existent or develops during fever, cardiac rhythm observation is indicated until the fever has subsided or the ECG has normalised. This should preferably be performed in a hospital with experience in the treatment of arrhythmias associated with BrS. If it is more convenient for the patient and their family, fever ECGs can also be recorded at the local hospital, which may be relevant in the current COVID-19 era [23]. However, a fever ECG should always be assessed by a paediatric cardiologist before making clinical decisions. Caution is advised in situations in which the body temperature can increase, such as while playing sports during a heatwave, or while taking a sauna or a steam bath.

Medication associated with an increased risk of arrhythmic events in BrS (see www.brugadadrugs.org) should be avoided. If the use of a particular drug is unavoidable, the development of a type 1 pattern due to the medication should be evaluated with ECG recordings before and after the drug intake. Lastly, the intake of excessive amounts of alcohol should be discouraged.

### Medication and other treatments

Ventricular arrhythmias or electrical storms in BrS are treated with isoproterenol infusion. Amiodarone and sodium channel blockers, which are advocated in advanced paediatric life support protocols, are contra-indicated in BrS patients. Monomorphic ventricular tachycardias during fever can be treated with a beta-blocker. Medical therapy with quinidine can be considered in highly symptomatic patients, but the evidence in children is limited [24, 25]. An implantable cardiac defibrillator should only be implanted according to international guidelines [26]. Although right ventricular outflow tract epicardial ablation seems promising in symptomatic adults with BrS [27], its short- and long-term effects in children are unknown. It can be considered in an experimental setting in children with uncontrollable ventricular arrhythmias.

## Limitations

Our expert consensus was limited, mainly due to the inclusion of retrospective cohort studies. Therefore, the level of evidence of this statement is low. Furthermore, not all possible BrS phenotypes could be described in detail in this article. However, this consensus agreement fills a gap and will likely improve standard of care for all children with BrS and those from BrS families.

## Supplementary Information

**Fig. S1** Standard 12-lead electrocardiogram showing Brugada type 1 pattern

**Fig. S2** Brugada electrocardiogram (*ECG*) in higher right precordial leads position. (This figure was adapted from an original figure from ©username:jmarchn/Wikimedia Commons/CC-BY-SA‑3.0 by adding the Brugada ECG leads position and legend)
