# Supplementary figures and images for: Screening, diagnosis and follow-up of Brugada syndrome in children: a Dutch expert consensus statement
Source: Neth Heart J. 2022 Oct 12;31(4):133–7. doi: 10.1007/s12471-022-01723-6 (PMC9554382; doi:10.1007/s12471-022-01723-6)

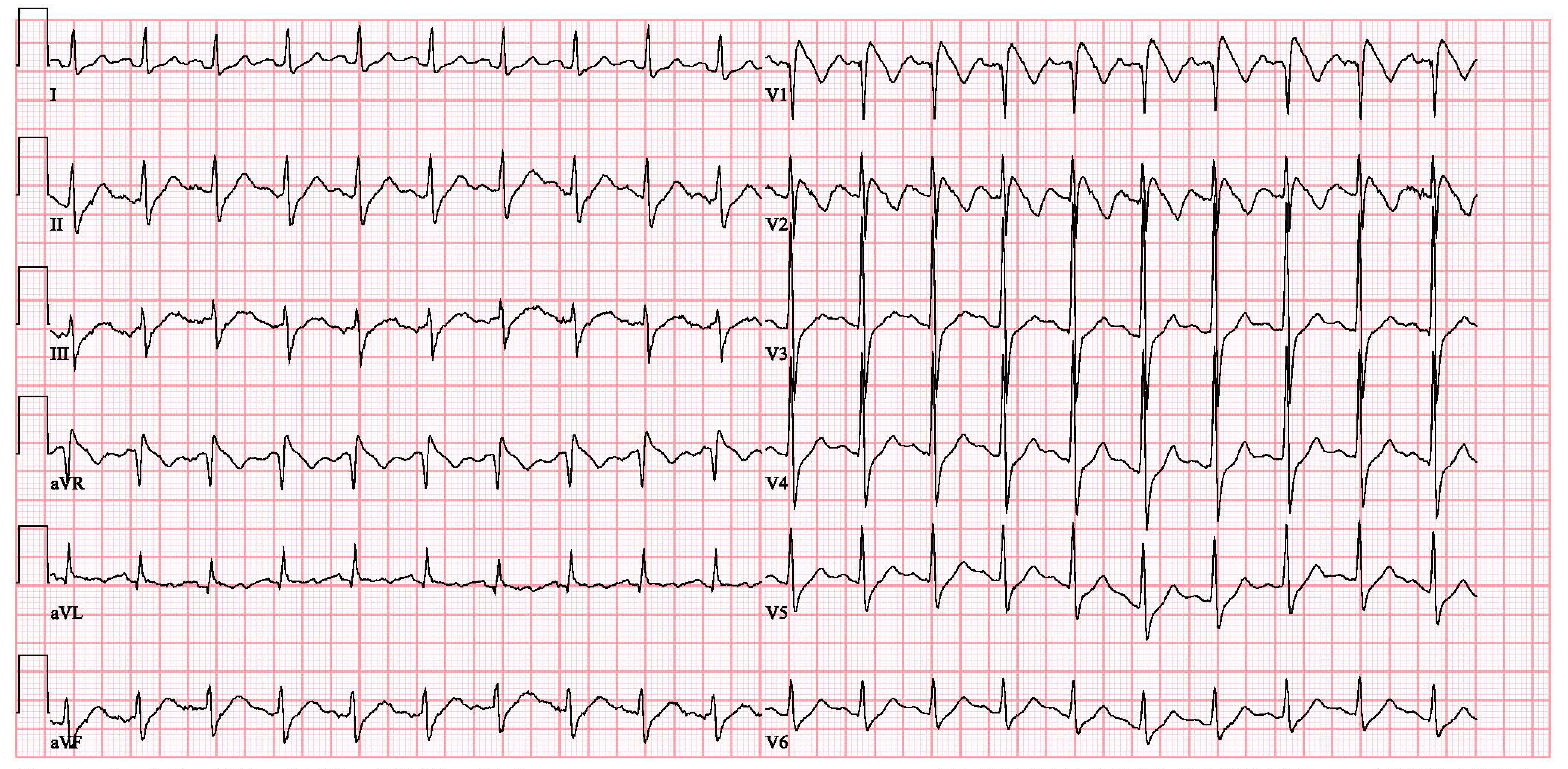

Supplement: Supplementary file 1 — Fig. S1 Standard 12-lead electrocardiogram showing Brugada type 1 pattern [file 12471_2022_1723_MOESM1_ESM.jpg]

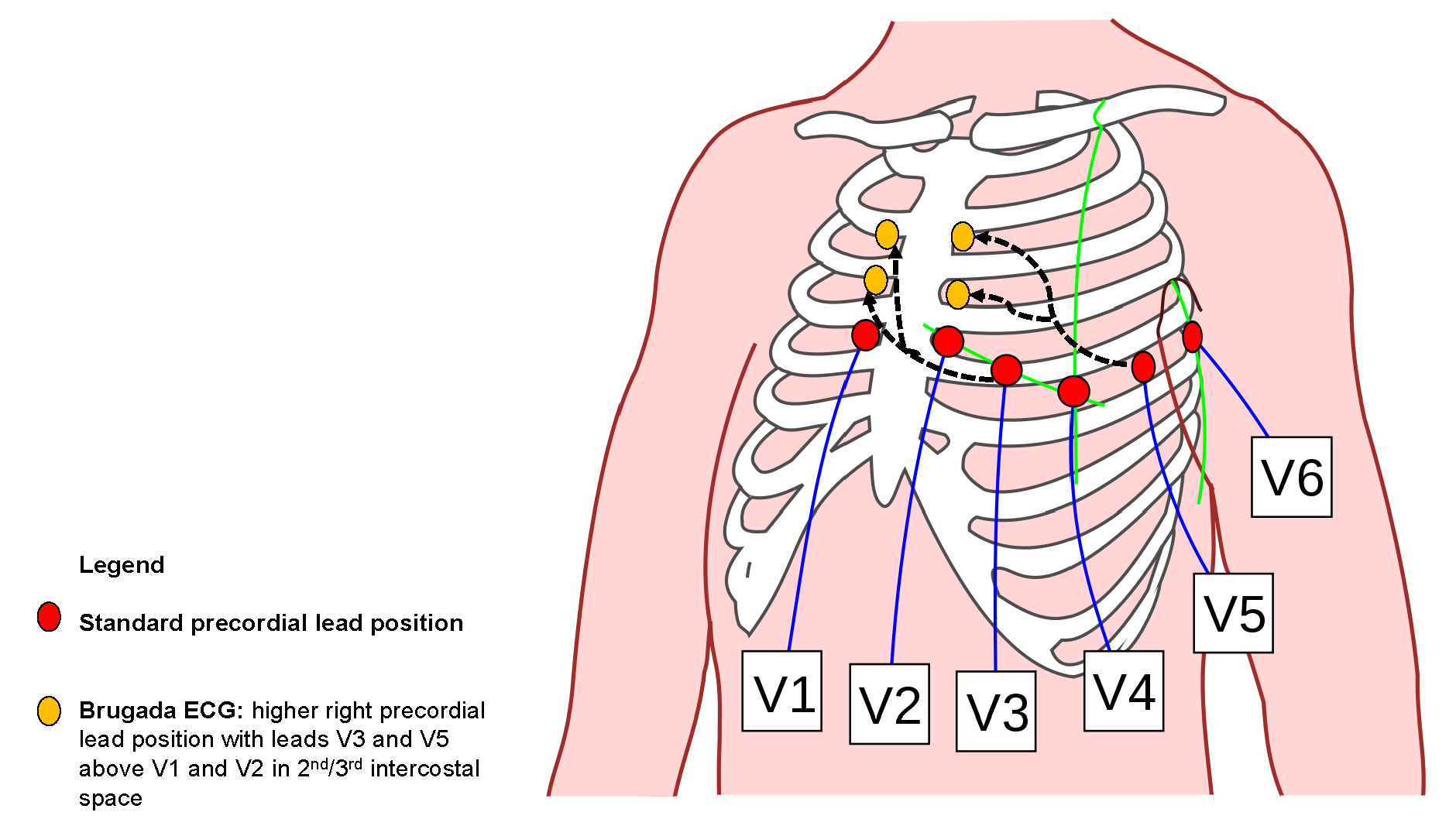

Supplement: Supplementary file 2 — Fig. S2 Brugada electrocardiogram (ECG) in higher right precordial leads position. (This figure was adapted from an original figure from ©username:jmarchn/Wikimedia Commons/CC-BY-SA‑3.0 by adding the Brugada ECG leads position and legend) [file 12471_2022_1723_MOESM2_ESM.tif]
